# Characteristics of open globe injury in children under six

**DOI:** 10.3389/fped.2024.1442531

**Published:** 2024-11-28

**Authors:** Rui Li, Shounan Qi, Ying Zhang, Chenguang Wang

**Affiliations:** ^1^ Department of Ophthalmology, The Second Hospital of Jilin University, Changchun, Jilin, China; ^2^ ShanXi Ophthalmic Hospital, Xian, Shanxi, China

**Keywords:** children, open globe injury, characteristics, endophthalmitis, amblyopia

## Abstract

**Introduction:**

This study aimed to analyse the clinical characteristics of open globe injury (OGI) in children under six.

**Methods:**

A retrospective analysis was conducted on the medical data of children with OGI admitted to the Eye Center of the Second Hospital of Jilin University, China, between 1 January 2012 and 31 December 2020.

**Results:**

The study included 106 children, with 61 males (57.5%) and 45 females (42.5%), and the mean age was 4.14 ± 1.58 years. Injuries predominantly affected the right eye (53.8%). Sharp objects were the leading cause of injuries (56.6%). Most wounds (91.5%) occurred in zone I, and penetrating trauma was the most common injury type (64.2%). The concomitant OGI findings included traumatic cataracts (47.2%), iris prolapse (53.8%), endophthalmitis (22.4%), vitreous prolapse (17.0%), retinal detachment (5.7%), eyelid laceration (7.5%), and orbital fractures (1.9%). Univariate analysis showed that wooden materials (*p* = 0.045), needles (*p* = 0.045), postinjury admission time (*p* < 0.001), injury zone (*p* = 0.025), and iris prolapse (*p* = 0.022) were significantly associated with endophthalmitis. Multivariate logistic regression analysis revealed that delayed admission for ≥24 h was an independent risk factor for endophthalmitis (*p* = 0.007). Preoperative and postoperative visual acuities were significantly correlated (*p* < 0.001).

**Conclusions:**

OGIs are more common in males under six years old, with sharp objects being the primary cause. Prompt surgical intervention can lower the risk of endophthalmitis. Increased awareness of the severity of OGI and understanding the risk factors for endophthalmitis will aid in developing effective strategies to minimise ocular complications.

## 1 Introduction

Ocular trauma is the leading cause of monocular visual disabilities and amblyopia in children (1). The global incidence of ocular trauma in children is 9–15 cases per 100,000, with open globe injury (OGI) accounting for 25% of these cases (2). Despite significant advancements in microsurgical techniques and a better understanding of OGI, postoperative outcomes in children often remain unsatisfactory (3). Aphakia, which results in loss of accommodation, and irregular astigmatism due to corneal scarring hinder postoperative visual rehabilitation and increases the risk of amblyopia in paediatric patients (4, 5). Previous research has identified that the age at injury (< six years) and the development of amblyopia are significant risk factors for poor outcomes in children with OGI (6, 7).

Although a previous study indicated a decrease in the annual incidence of paediatric eye injuries (8), preschool children are still more susceptible to OGI compared to school-aged children owing to their less coordinated motor skills and lack of self-protection awareness (3, 9, 10). Additionally, insufficient knowledge about emergency treatment further increases the risk of visual impairment and complications. From the perspective of public health and eye injury prevention, identifying the injury mechanism, types, management, and prognosis of OGI is crucial for effective health education and the development of preventive measures.

The United Nations Convention on the Rights of the Child defines children as individuals aged 0–18 years (11). However, most previous studies on OGI have focused on older children, leaving a gap in research specifically addressing children under six years old (12–14). Gunes found that in Turkey, OGI in children under six years of age predominantly occurs at home, with kitchen items being the most common cause of injury (15). Read et al. (7) identified glass as the leading cause of injury and highlighted unique risk factors for poor outcomes in children under six with OGI. Endophthalmitis is a severe and potentially devastating complication; however, studies on posttraumatic endophthalmitis in children are limited (5, 16). This study aimed to analyse the clinical characteristics of OGI in children under six years and identify risk factors for endophthalmitis following OGI, providing updated evidence in this field.

## 2 Materials and methods

### 2.1 Statement of ethics

This population-based retrospective study involved children with OGI confirmed in the operating room of the Eye Center of the Second Hospital of Jilin University, China, between 1 January 2012 and 31 December 2020. The Ethics Committee of the Second Hospital of Jilin University approved the study, which adhered to the ethical standards of the 1964 Declaration of Helsinki and its subsequent amendments. The requirement for written informed consent was waived due to the study's retrospective nature.

### 2.2 Patient selection

All children under 6 years admitted to the hospital, including ED admissions, were included. The exclusion criteria were as follows: (1) age over six years; (2) closed globe injuries, children with complicating factors such as thermal and chemical injuries; and (3) incomplete medical records (seven patients with missing clinical data who opted for secondary surgery at other hospitals after initial surgical repair).

### 2.3 Data collection

Data recorded for eligible OGI children included age, sex, injury mechanism (knives, glass, wooden materials, blunts, needles, corn stalks), the interval between the injury occurrence and hospital admission, clinical signs, OGI type [penetrating injury, perforating injury, intraocular foreign body (IOFB), and globe rupture], and wound location (zone I: full-thickness injury limited to the cornea; zone II: full-thickness injury within 5 mm posterior to the corneoscleral limbus; zone III: full-thickness injury posterior to zone II). Trauma types and injury zones were defined according to the Birmingham Eye Trauma Terminology and Eye Trauma Classification Group Guidelines (17). All admitted patients underwent detailed ophthalmological examinations performed by an operator with at least 10 years of clinical experience. Patients were asked about any history of refractive error, and posttraumatic presenting visual acuity (VA) and the best-corrected VA at the final follow-up were recorded whenever possible using the Snellen chart, considering the challenges of obtaining cooperation from young children. The follow-up time was 6–17 months (average, 9.01 ± 0.31 months).

### 2.4 Statistical analysis

Data analysis was performed using SPSS version 26.0 (IBM Corporation, Armonk, NY, USA; https://www.ibm.com). Categorical variables were expressed as frequencies and percentages, whereas continuous variables were expressed as means (standard deviation). Univariate analyses were conducted for age, sex, zone, post-injury admission time, IOFB, injury material, traumatic cataract, and iris prolapse to assess risk factors for endophthalmitis. A rank-sum test was used to explore the differences between continuous variables, such as age. The Chi-square or Fisher's exact test was used for categorical data (sex, zone, post-injury admission time, IOFB, injury material, traumatic cataracts, and iris prolapse). Variables associated with endophthalmitis were included in a multiple logistic regression model to identify independent predictors of endophthalmitis. A *p*-value < 0.05 was considered statistically significant.

## 3 Results

### 3.1 Clinical features

**Figure 1:**
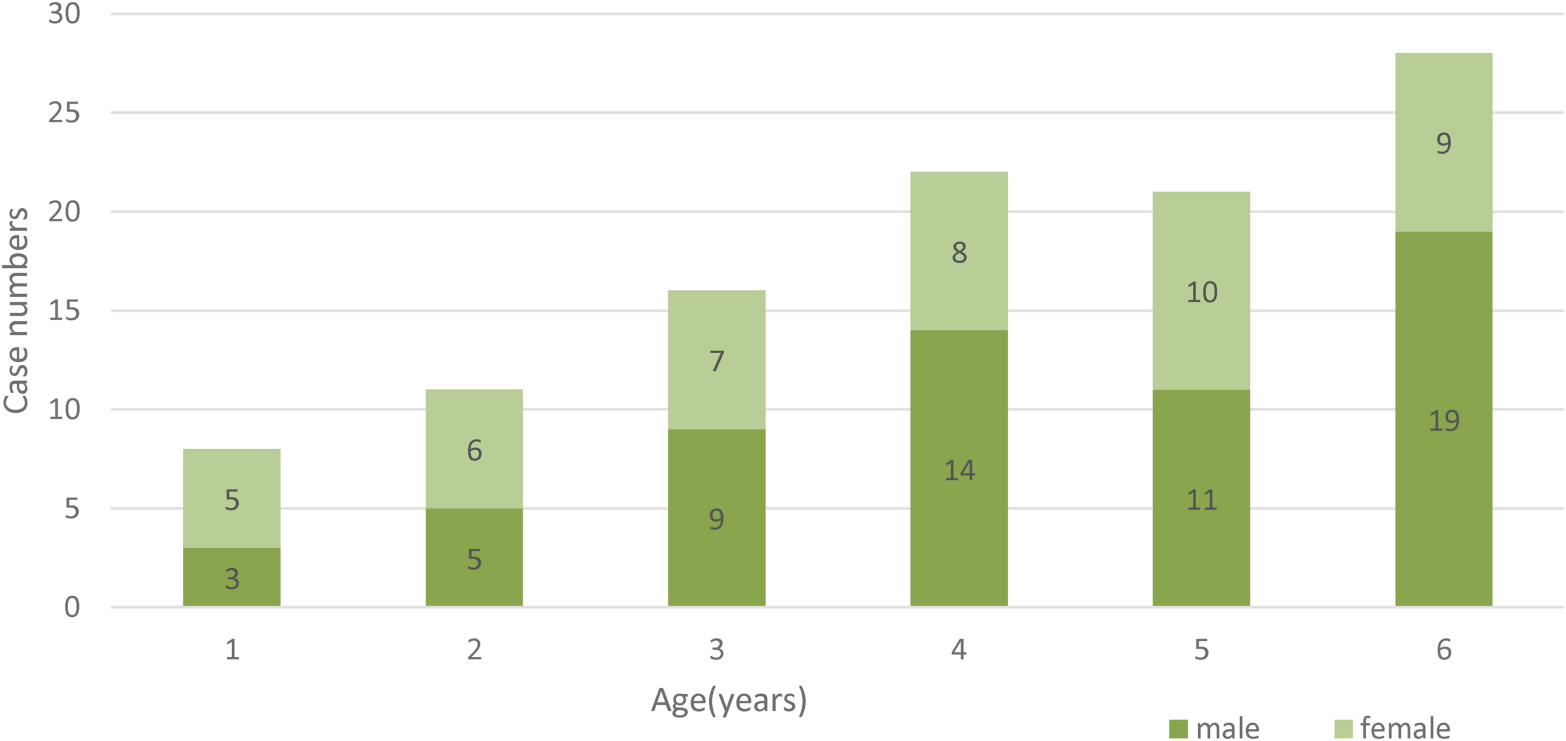
Age and gender distribution of children under 6 years with OGI.

This study included 106 patients. Table 1 shows the clinical features of all the patients. The mean age was 4.14 ± 1.58 years. The prevalence of OGI was higher in males (61 cases, 57.5%) than in females (45 cases, 42.5%). Most traumas were caused by sharp objects (56.6%). The age and sex distributions of the OGI are shown in Figure 1.

**Table 1:** Clinical features of open globe injuries in children (*n* = 106).

Characteristics	Group	Number	(%)
Age (years)	0–1	8	7.5%
	1–2	11	10.4%
	2–3	16	15.1%
	3–4	22	20.8%
	4–5	21	19.8%
	5–6	28	26.4%
Gender	Male	61	57.5%
	Female	45	42.5%
Affected eye	Left	49	46.2%
	Right	57	53.8%
Injury type	Penetration	68	64.2%
	Rupture	15	14.2%
	IOFB	13	12.3%
	Perforation	10	9.4%
Post-injury admission time (h)*	<24	61	59.2%
	≥24	42	40.8%
Zone	Zone I	73	68.9%
	Zone II	5	4.7%
	Zone III	1	0.9%
	Multiple zones	27	25.5%
Injury material	Knives	22	20.8%
	Glass	18	17.0%
	Blunts	11	10.4%
	Needles	10	9.4%
	Wooden materials	10	9.4%
	Toys	10	9.4%
	Others	9	8.5%
	Stationery	8	7.5%
	Corn stalks	4	3.8%
	Firecrackers	4	3.8%
Concomitant diagnosis	Iris prolapse	57	53.8%
	Traumatic cataracts	50	47.2%
	Endophthalmitis	24	22.4%
	Vitreous prolapse	18	17.0%
	Eyelid laceration	8	7.5%
	Retinal detachment	6	5.7%
	Orbital fractures	2	1.9%

Zone I, cornea and limbus; Zone II, 5 mm posterior to the limbal; Zone III, posterior sclera *Excluding 3 cases with unclear time of injury.

Among the patients, 61 (59.2%) were admitted within 24 h of injury, 42 (40.8%) were admitted after 24 h, and the time of admission was not available for three patients. Penetrating injury was the most common type of OGI (68 patients, 64.2%). Zone I injuries occurred in 68.9% of the patients, whereas 4.7% and 0.9% had injuries in zones II and III, respectively. A total of 27 (25.5%) children had injuries in multiple zones, with 18 (17.0%) in zones I and II, three (2.8%) in zones II and III, and six (5.7%) in all zones.

### 3.2 Surgery details

All children under six years of age with OGI underwent primary surgical repair. Traumatic cataracts (47.2%) were the most common concomitant injuries. Among these children, 26.0% underwent primary cataract extraction due to anterior capsule rupture and cortical overflow, whereas 6% underwent treatment with secondary lens extraction after the resolution of corneal oedema and inflammation. Nineteen patients (17.9%) underwent intraocular lens implantation. Intraoperatively, 70 patients (66.0%) with OGI received intravitreal injections of vancomycin, and 18 (17.0%) underwent secondary pars plana vitrectomy.

### 3.3 Analysis of factors associated with endophthalmitis

A total of 24 patients (22.4%) were clinically diagnosed with endophthalmitis. Univariate analyses for age, sex, zone, post-injury admission time, IOFB, injury material, traumatic cataract, and iris prolapse were conducted to assess the risk factors for endophthalmitis (Table 2). The results showed that injuries caused by needles and wood materials were statistically associated with endophthalmitis (*p* = 0.045). Additionally, post-injury admission time (*p* < 0.001), injury zone (*p* = 0.025), and iris prolapse (*p* = 0.022) were significantly associated with endophthalmitis. These statistically relevant variables were included in a multivariate logistic regression analysis (Table 3), revealing that delayed admission (≥24 h) was an independent risk factor for endophthalmitis (*p* = 0.007; OR = 4.620; 1.524, 14.004). Only one of the 24 endophthalmitis cases showed a positive organism culture, which was a case of Streptococcus spp in vitreous cultures.

**Table 2:** Univariate analysis: presentation features associated with the development of endophthalmitis.

Parameter	Teams	Endophthalmitis		*p* value
		Yes	No	
Mean age ± SD		3.88 ± 0.32	4.22 ± 0.17	0.382
Sex	Male	14 (58.3%)	47 (57.3%)	0.929
	Female	10 (41.7%)	35 (42.7%)	
Injury type	Penetration	13 (54.2%)	55 (67.1%)	0.177
	Perforation	4 (16.7%)	6 (7.3%)	
	Rupture	2 (8.3%)	13 (15.9%)	
	IOFB	5 (20.8%)	8 (9.8%)	
Zone	Zone I	21 (87.5%)	52 (63.4%)	0.025
	Zone I and zone II/III	3 (12.5%)	30 (36.6%)	
Post-injury admission time (h)	<24	6 (25.0%)	55 (69.6%)	<0.001
	≥24	18 (75.0%)	24 (30.4%)	
IOFB	Yes	5 (20.8%)	8 (9.8%)	0.164
	No	19 (79.2%)	74 (90.2%)	
Injury Material	Knives	4 (16.7%)	18 (22.0%)	0.776
	Glass and porcelain fragments	2 (8.3%)	16 (19.5%)	0.166
	Wooden materials	5 (20.8%)	5 (6.1%)	0.045
	Needles	5 (20.8%)	5 (6.1%)	0.045
	Corn stalks	1 (4.2%)	3 (3.7%)	0.648
	Firecrackers	1 (4.2%)	3 (3.7%)	0.648
	Toys	4 (16.7%)	6 (7.3%)	0.229
	Stationery	1 (4.2%)	7 (8.5%)	0.680
	Blunts	1 (4.2%)	10 (12.2%)	0.450
	Others	0 (0%)	9 (11.0%)	0.204
Traumatic cataracts	Yes	14 (58.3%)	36 (43.9%)	0.213
	No	10 (41.7)	46 (56.1%)	
Iris prolapse	Yes	8 (33.3%)	49 (46.2%)	0.022
	No	16 (66.7%)	33 (40.2%)	

IOFB, intraocular foreign body.

**Table 3:** Multivariate logistic regression analysis of endophthalmitis.

Clinical feature	Odds ratio	95%CI		*P*-value
		Lower	Upper	
Needles	3.321	0.766	14.211	0.106
Wooden materials	3.462	0.731	16.395	0.118
Post-injury admission time (h)	4.620	1.524	14.004	0.007
Iris prolapse	0.403	0.137	1.185	0.099
Zone	3.141	0.747	13.207	0.118

### 3.4 Visual acuity results

In this study, all patients who were followed up had varying degrees of visual function salvage (VA ranging from light perception to 20/25 after surgery), and none underwent eye removal surgery during the follow-up period. Two patients (1.8%) had myopia before OGI, and no patient was wearing refractive error glasses at the time of injury. Preoperative and postoperative VA were obtained from 41 patients. VA could not be measured in other patients because they were too young or unable to cooperate with VA testing. The initial and final VA values at the final follow-up are presented in Table 4. In the study, preoperative and postoperative VA showed a statistically significant correlation (*p* < 0.001).

**Table 4:** Comparison of the initial VA and final BCVA at the last follow-up.

VA	≥20/40	20/50–20/100	19/100–5/200	4/200-LP	NLP
Initial VA	3	6	8	22	2
Final BCVA	13	10	8	10	0

VA, visual acuity; BCVA, best-corrected visual acuity; LP, light perception; NLP, no light perception.

## 4 Discussion

The study revealed that boys experience a significantly higher rate of OGI compared to girls at the age of six. This finding contrasts with those of previous studies on paediatric OGI (3, 5, 18, 19). We attribute this disparity to the fact that, at younger ages, boys and girls typically engage in similar activities. However, as they grow older, boys are more likely to handle sharp objects, such as toys, which increases the risk of accidental eye injuries. Sharp objects are a major cause of OGI in children (48%–75%) (6, 18, 20, 21) – a finding that was corroborated by our study, which identified sharp objects as causing 56.6% of OGI cases. This result aligns with that of AIDahash et al. (18), who reported that knives were the leading cause of eye injuries in children. Conversely, Read and Cavuoto (7) concluded that preschool injuries were more frequently caused by glasses. In previous studies, injuries in zone I were more common than those in other zones, and multizone injuries were rare (3, 5, 12, 15, 19, 21); however, these accounted for 25% of all cases in the present study. This could be attributed to the difficulty young children face in protecting themselves from hazardous objects.

Assessment of OGI in children presents challenges owing to incomplete or inaccurate injury descriptions and difficulties in obtaining cooperation during ocular examinations. Therefore, the primary treatment may be delayed, increasing the risk of complications. Traumatic cataract was the most common concomitant finding, occurring in 47.2% of cases. Additionally, 19 patients with aphakia chose not to undergo lens implantation, as further surgery was unlikely to improve their postoperative vision. Posttraumatic endophthalmitis is one of the most severe complications (22). The incidence of endophthalmitis in this study was 22.4%, this is consistent with previous studies where the incidence rates in children ranged from 13% to 54% (5, 19, 23, 24). Studies on OGI have demonstrated that delayed wound repair (22), IOFB presence (25, 26), dirty wounds (25), posterior capsule rupture (22), and trauma occurring in rural or outdoor areas (16, 23) were associated with an increased risk of posttraumatic endophthalmitis. The incidence of endophthalmitis in hospitalised patients without associated risk factors was 5.9%, which increased to 65.3% and 90.3% when patients were exposed to two and three risk factors, respectively (25).

As demonstrated in this study, needles and wood were the most common materials causing injuries in endophthalmitis. Therefore, ophthalmologists must try to elicit as many details as possible regarding the mechanism of injury and take measures to prevent endophthalmitis when treating OGI, such as expediting surgical repair and injecting intravitreal antibiotics. In our study, wounds involving only zone I were more common than those involving other zones. In a study from India (16), the risk of endophthalmitis induced by a central corneal wound (central 5 mm) was nine times higher than that caused by peripheral corneal wounds. This was probably because it was difficult for the torn cornea to close smoothly in the early stage, in which case contaminating microorganisms remained in the ocular cavity to replicate. Additionally, the avascular nature of the corneal results in an insufficient immune response and delayed wound healing (5, 16).

Bansal et al. (22) and Zhang et al. (23) found that intraocular tissue prolapse played a protective role in reducing the risk of endophthalmitis in children. They suggested that prolapsed tissue could seal the wound and prevent the entry of microorganisms. However, a recent study (16) showed that wound-induced iris and vitreous prolapses increased the probability of endophthalmitis in children by three and five times, respectively. Our univariate analysis indicated that iris prolapse was associated with a decreased risk of infection. Larger studies are needed to further analyse whether intraocular tissue prolapse promotes or prevents a microbial invasion.

The most critical approach to preventing posttraumatic endophthalmitis in children is early hospital admission for ocular repair and the use of prophylactic intravitreal antibiotics (22). In this study, all children diagnosed with OGI underwent primary repair surgery. Intravitreal vancomycin injections were administered to 66.0% of patients, whereas systemic antibiotics were administered to the remaining patients owing to mild wound inflammation. Logistic regression analysis showed that Delayed admission of ≥24 h was an independent risk factor. This result is consistent with those of previous studies on paediatric OGI (10, 23–25).

The VA results were limited due to the young age of the study participants. Initial VA was an important determinant of final VA, consistent with previous results (12, 18, 21). Notably, eye trauma in children can increase the risk of amblyopia. One study reported that OGI was associated with two major refraction-related complications: loss of accommodation due to aphakia and irregular astigmatism caused by corneal scarring (4). These complications can be managed with contact lenses, spectacles, or surgical treatments (e.g., corneal transplantation) to reduce the risk of amblyopia. Additionally, regular follow-ups and BCVA monitoring are equally important. A specialist ophthalmology team administers amblyopia treatment and refractive correction to facilitate visual recovery. The use of the amblyopic eye can be facilitated by preventing visual input from the better eye once the patient's best-corrected VA decline is identified.

This study had some limitations. It was a retrospective study; hence, certain risk factors could not be assessed, as the children could not disclose all the details of the injury. Additionally, patients treated at other hospitals were excluded.

## 5 Conclusions

In children under the age of six, OGI occurred more frequently in males than in females, with sharp objects being the primary cause. Delayed admission of ≥24 h is an independent risk factor for children with endophthalmitis. Awareness of the seriousness of OGI in children and understanding the risk factors for endophthalmitis will help develop effective strategies to reduce the incidence of ocular complications.

## Data Availability

The raw data supporting the conclusions of this article will be made available by the authors, without undue reservation.
